# Wide-Range, Washable Piezoresistive Pressure Sensor Based on MCNT-PDMS Dip-Coated PDMS Sponge

**DOI:** 10.3390/mi16040477

**Published:** 2025-04-17

**Authors:** Kun Luo, Xinyi Wang, Tao Xue, Yingying Zhao, Qiang Zou

**Affiliations:** 1 School of Microelectronics, Tianjin University, Tianjin 300072, China; 2022232152@tju.edu.cn; 2 Tianjin Flying Pigeon Group Co., Ltd., Tianjin 301600, China; 13116091576@163.com; 3 Center of Analysis and Testing Facilities, Tianjin University, Tianjin 300072, China; xuetao@tju.edu.cn; 4 Tianjin International Joint Research Center for Internet of Things, Tianjin 300072, China; 18622912991@163.com; 5 Tianjin Key Laboratory of Imaging and Sensing Microelectronic Technology, Tianjin University, Tianjin 300072, China; 6 State Key Laboratory of Advanced Materials for Intelligent Sensing, Tianjin University, Tianjin 300072, China

**Keywords:** flexible pressure sensors, carbon nanotube, PDMS sponge, wide-range pressure sensing, piezoresistive sensor, washable

## Abstract

Flexible pressure sensors have great potential for wearable applications such as human health monitoring and human–computer interaction, which require different trade-offs between the sensitivity and operating range. However, preparing washable and wide-range piezoresistive pressure sensors remains a great challenge. Here, we developed a porous flexible elastomer sponge based on a carbon nanotube composite network coating for pressure sensors with extremely high stability and washability over a wide range. Specifically, a sugar template was used to fabricate a homogeneous macroporous PDMS sponge as a substrate, and a dip-coated MCNT-PDMS composite was used as a conductive layer. The high degree of adhesion formed between the substrate and the conductive layer resulted in a sponge with greatly enhanced mechanical properties and stability, while improving the operating range. The pressure sensors exhibited a broad operating range of 0–650 kPa, demonstrating excellent sensitivity (0.0049 kPa^−1^ in the range of 0–74 kPa, 0.0010 kPa^−1^ in the range of 74–310 kPa, and 0.0004 kPa^−1^ in the range of 310–650 kPa), as well as a fast response time of 143 ms and recovery time of 73 ms, long-term cycling stability of over 10,000 cycles, and excellent washable stability. Finally, we demonstrate that the sensors can be applied to gesture monitoring, human motion gait monitoring, and cycling pressure monitoring.

## 1. Introduction

In recent years, flexible pressure sensors have garnered significant attention due to their vast potential in applications such as wearable medical devices [1,2,3,4], human motion monitoring [5,6], and human–computer interaction [7,8]. Their flexibility, sensitivity, durability, and excellent biocompatibility [9] make them particularly appealing for these uses. Flexible pressure sensors can be classified into three types based on their working mechanisms: piezoresistive, piezoelectric, and capacitive sensors. Among these, piezoresistive pressure sensors have been the most extensively studied due to their advantages, including simple fabrication, low costs, ease of signal acquisition, a rapid response, and a broad detection range.

Flexible piezoresistive pressure sensors are generally fabricated by embedding conductive materials, such as carbon black or carbon nanotubes, into a polymer or elastomer matrix (e.g., polydimethylsiloxane (PDMS), Ecoflex, or polyurethane). However, these sensors often suffer from low sensitivity and poor hysteresis over a wide range of pressures, primarily due to the imperfect recovery of the conductive nanomaterial network and the inherent viscoelasticity of the matrix material. This limits their practical applications [10]. To address this issue, micro-/nanostructured pressure sensors have emerged as a promising strategy to enhance sensor performance [11,12,13]. Micro-/nanostructures, such as domes [14], pyramids [15,16,17], wrinkles [18,19,20], bionics [21,22,23], and porous designs [24,25,26], exhibit easy deformability, which improves the sensing performance. Jihun Lee et al. [27] developed concentric circle pattern (CCP) piezoresistive pressure sensors using the fused deposition modeling (FDM) 3D printing technique, demonstrating high sensitivity (160 kPa^−1^) over a linear pressure range of 0–0.577 kPa. Sanghun Shin et al. [28] employed the same method to prepare crack-based strain gauge sensors, which formed self-aligned crack arrays during stretching, resulting in a 442% increase in the strain coefficient, a 99% reduction in the recovery time, and superior stability compared to planar sensors. These sensors demonstrate high sensitivity, a rapid response, and good stability. However, due to the limited deformation of micro-/nanostructures, their measurable pressure range remains small, making them unsuitable for applications requiring a wide dynamic range. Additionally, the fabrication of these microstructures typically involves complex and costly processes [29,30], hindering large-scale production.

Porous structure-based flexible pressure sensors have attracted considerable interest due to their simple fabrication methods and wide sensing ranges [31,32]. These sensors often use porous sponges coated with conductive materials like graphene, carbon nanotubes, or carbon black through techniques such as dip coating [33,34]. This approach ensures stable performance and broad sensitivity. The main challenges in dip coating conductive “inks” on stencil sponges include the instability and inhomogeneity of the conductive ink, weak physical interactions (e.g., electrostatic forces, π–π interactions), and the poor adhesion of the conductive coating material to the stencil. The aggregation of carbon or metal nanomaterials in suspension can hinder the formulation of stable, homogeneous “inks” and the uniform coating of templates. Additionally, poor adhesion can cause carbon or metal nanomaterials to rupture and peel off from the sponge. The wettability of the sponge and the conductive composition must be carefully considered to achieve a uniform and robust conductive coating on the stencil sponge. In this context, conductive polymers may offer a promising solution due to their softness and homogeneous dispersion, which allows for uniform dip coating and strong adhesion [35,36,37]. Polymers of the same composition have strong interactions with each other due to the mutual diffusion of polymer molecules at the interface [38,39]. This makes it a feasible strategy to construct conductive polymer coatings with the same composition as the template sponge. PDMS has been widely used as a sponge substrate due to its good biocompatibility and the low-cost templating methods based on salts [40] or sugars [41]. While these methods can be simple and efficient for the preparation of template sponges, they have the disadvantage of producing irregular sponge shapes. The micropores in the prepared sponges are often disordered and vary in size, leading to issues such as the curing of the conductive polymer coating in smaller micropores, as well as blockage and uneven coating caused by the unfavorable flow of the coating, which greatly affects sensors’ performance. However, the use of polymer bead stacking [42], which ensures spatial uniformity and a consistent bead size, can result in a sponge with uniformly arranged micropores of the same size. This method, although effective, often requires a more complex process.

To address the aforementioned issues, this paper proposes a PDMS porous sponge pressure sensor prepared using a template sacrifice method based on uniformly arranged sucrose pills and coated with MCNT-PDMS. Since the micropores of the PDMS sponge are nearly uniform in size and evenly distributed, the MCNT-PDMS coating can effectively penetrate the PDMS sponge. By using the same composition for both the coating and the sponge substrate, the strong adhesion between the MCNT-PDMS coating and the sponge skeleton enhances the mechanical properties and stability of the sensors. This allows the sensors to operate effectively across a pressure range of up to 650 kPa. Furthermore, because PDMS is inherently hydrophobic and the coating does not clog the sponge’s micropores, the sensors exhibit excellent washability and sufficient sensitivity over a wide pressure range. Extensive cycling tests (over 10,000 cycles) have demonstrated the excellent stability and repeatability of the sensors. These PDMS sponge-based pressure sensors with MCNT-PDMS coatings show great potential for real-time human health monitoring and cycling pressure monitoring.

## 2. Experimental Details

### 2.1. Materials

The materials used for the fabrication of the carbon nanotube composite network-coated PDMS sponges included carbon nanotubes (purchased from Chengdu Jiazai Science and Technology Co., Ltd., Chengdu, China, purity > 98%, diameter 10–30 nm, length 10–20 μm), isopropyl alcohol (IPA) (obtained from Shanghai McLean Biochemical Science and Technology Co., Ltd., Shanghai, China, anhydrous, 99.9%), sucrose sugar pills (sourced from Zhejiang Haining Weijing Pharmaceutical Excipients Technology Development Co., Ltd., Haining, China, diameter 1–1.18 mm), and a colloidal PDMS polymer with a curing agent (Sylgard 184, Dow Corning, Inc., Hemlock, MI, USA).

### 2.2. Preparation of Porous PDMS Sponges

Sucrose sugar pills were placed into a 2 cm × 1 cm × 2 cm plastic mold and shaken to ensure that the pills filled the voids, and a 2 cm × 1 cm plastic press with the corners ground off was used to press the pills to a thickness of 7 mm and held in place with a clamp. PDMS was prepared by mixing the PDMS prepolymer with the crosslinking curing agent in a weight ratio of 10:1. A vacuum dryer was used to remove air bubbles from the PDMS mixture. The PDMS liquid was then poured into the mold and infiltrated under a vacuum for 1 h to allow the pores between the sugar pills to be filled with the PDMS colloid. Afterward, the molds were placed in an oven and cured at 70 °C for 3 h. The resulting blocks were then placed in a hot water bath at 100 °C for 2 h and repeatedly squeezed in warm water at 60 °C to dissolve the sugar pills, leaving behind the PDMS sponge.

### 2.3. Preparation of Carbon Nanotube Composite Network-Coated PDMS (MCNT-PDMS/ PDMS) Sponges

Multi-walled carbon nanotubes (MCNTs) were dispersed in isopropanol, vortex-mixed for 1 h, and sonicated for 1 h to prepare a homogeneous MCNT-IPA dispersion. Uniform MCNT-PDMS liquids with varying mass ratios (4 wt%, 5 wt%, 6 wt%, 7 wt%, 8 wt%) were prepared by adding different amounts of the MCNT-IPA dispersion into the PDMS prepolymer. The mixture was sonicated for 1 h and stirred with a magnetic stirrer at 70 °C for 1 h to remove the isopropanol. A crosslinking curing agent was then added at a weight ratio of 10:1 to the PDMS prepolymer. The porous PDMS sponge was immersed in the MCNT-PDMS liquid and repeatedly squeezed to coat the main chain surface of the PDMS with MCNT-PDMS. The excess MCNT-PDMS liquid was then wiped off with oil-absorbent paper to prevent the clogging of the pores. Finally, the sponge was placed in an oven at 70 °C for 2 h to cure, resulting in PDMS sponges coated with different mass ratios of MCNT-PDMS.

### 2.4. Preparation of MCNT-PDMS/PDMS Sponge Pressure Sensors

To prevent signal drift, conductive nonwoven fabrics were attached to both the upper and lower surfaces of the MCNT-PDMS/PDMS sponges using conductive silver paste.

### 2.5. Characterization

The morphologies of the pure PDMS sponges and MCNT-PDMS/PDMS sponges were examined using scanning electron microscopy (SEM). The sensor resistance was measured with a digital multimeter (KEITHLEY, 2110, Keithley Instruments, Inc., Solon, OH, USA). Specific pressure was applied using a force gauge (AIGU-ZP-100, ETOOL Co., Ltd., Tokyo, Japan) combined with a self-assembled high-precision displacement stage (42BYG4812AA, Jiangsu Huisitong Co., Ltd., Wuxi, China). The test system consisted of a computer, a digital multimeter, and a pressure system. The pressure system applied pressure to the transducer in the vertical direction, while the digital multimeter and computer software were responsible for measuring and recording data, respectively. For cyclic strain testing, the transducer was fixed to the test system, and a specific strain was repeatedly applied and released while an electrical signal was recorded. The mechanical properties of the pure PDMS sponges and MCNT-PDMS/PDMS sponges were tested using a mechanical testing machine (KY-1KNW, Shanghai Kaiyan Testing Instruments Co., Ltd., Shanghai, China). At least five samples were tested to evaluate the mechanical and sensing properties, and the average results are reported.

## 3. Results and Discussion

**Figure 1:**
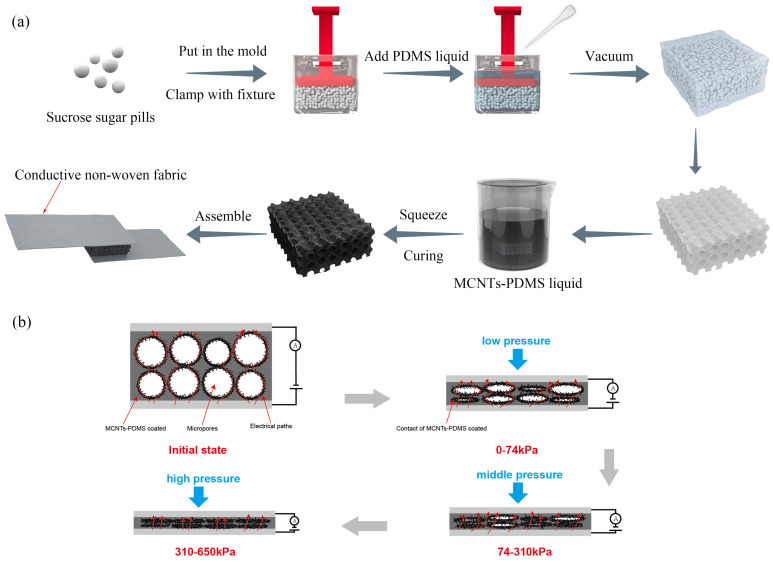
(**a**) Preparation process of the MCNT-PDMS/PDMS sponge pressure sensor. (**b**) Schematic diagram of the pressure sensing mechanism of the MCNT-PDMS/PDMS sponge pressure sensor.

Figure 1a illustrates the preparation of the sponge sensors through a simple process involving mixing, dissolving, dip coating, drying, and assembling. Since the sugar pills were nearly uniform in size and confined within the plastic mold, they were neatly and tightly arranged after oscillation. As a result, the micropores of the PDMS sponges fabricated using these sugar pill templates were nearly uniform in size and evenly distributed. Additionally, the fluidity of the MCNT-PDMS coatings was improved by the presence of a small amount of IPA, which facilitated the penetration of the MCNT-PDMS mixture into the PDMS sponge and ensured uniform adhesion to the sponge’s structure. The conductive layer was coated with MCNT-PDMS, which had the same composition as the PDMS sponge, ensuring strong adhesion between the coating and the porous network. To maintain the integrity of the micropores and prevent the curing of the MCNT-PDMS coating, the excess MCNT-PDMS solution was removed using blotting paper after the PDMS sponge was sufficiently immersed in the coating. This step contributed to the preparation of pressure sensors with excellent washable stability, a wide pressure range, and sufficient sensitivity. Figure 1b shows a schematic diagram of the pressure sensing mechanism of the MCNT-PDMS/PDMS sponge pressure sensor. Initially, the current flows primarily through the contact of MCNTs between different micropores, forming a conductive pathway (indicated by red arrows). Under low-pressure loading, the micropores with smaller diameters gradually close, causing the coatings on the inner walls of the micropores to come into contact, which creates additional conductive paths and leads to a decrease in the resistance of the MCNT-PDMS sponge pressure sensors. As the pressure increases to a medium level, the larger micropores also begin to close, resulting in a further decrease in the sensor’s resistance. With a further increase in pressure, all the micropores of the sponge pressure sensor close, causing the MCNT-PDMS coating on the inner walls to compress. As the pressure continues to rise, the MCNTs embedded within the coating make contact with each other, further reducing the sensor’s resistance.

**Figure 2:**
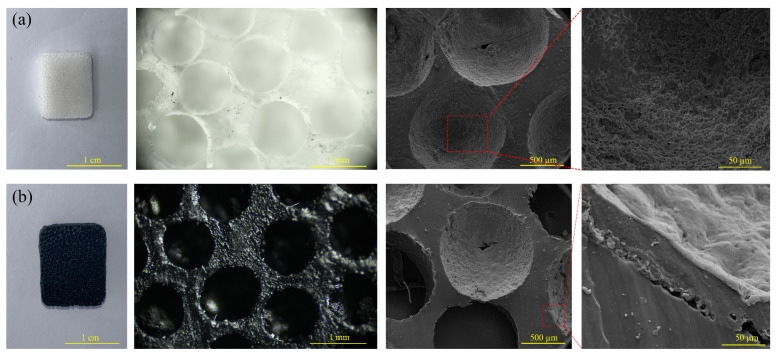
Photographs, optical microscopy images, and cross-sectional SEM images of the sponge sensors: (**a**) pure PDMS sponge; (**b**) MCNT-PDMS/PDMS sponge.

Figure 2 shows photographic, optical microscopy, and scanning electron microscopy (SEM) images of the PDMS and MCNT-PDMS/PDMS elastomer sponges. The pure PDMS sponge, as seen in Figure 2a, exhibits a white appearance, indicating the scattering of visible light due to its uniform porous structure. Under the optical microscope, the sponge’s pores are observed to be uniform in size and distribution, with a smooth surface. The roughness observed on the sponge surface may be attributed to the uneven surface of the sugar pills when the sample cross-section is viewed under SEM. In contrast, the MCNT-PDMS/PDMS sponges in Figure 2b appear black. Further examination under the light microscope reveals that the PDMS sponges are uniformly coated with MCNT-PDMS on the surface. As shown by the SEM images, the micropores are not significantly different in morphology compared with pure PDMS, while a thin layer with insignificant demarcation is found smooth on the inner surface boundary of the pores, indicating that the MCNT-PDMS coating is evenly distributed inside the PDMS sponge without blocking the apertures, thereby forming a stable conductive pathway. Further observation at the interface reveals that the coating and the surfaces of the holes are fused at certain locations with close contact, suggesting strong adhesion between the PDMS molecules in the coating and the sponge skeleton due to mutual diffusion, facilitated by the fact that both the coating and the sponge have the same composition. The conductive polymer coating not only enhances the mechanical strength of the large-aperture sponge but also provides a strong adhesive conductive layer, ensuring high stability and a wide operating pressure range for the sensors, even under frequent deformation. The hydrophobic properties of the cured PDMS contribute to the excellent washable stability of the sponge sensors.

**Figure 3:**
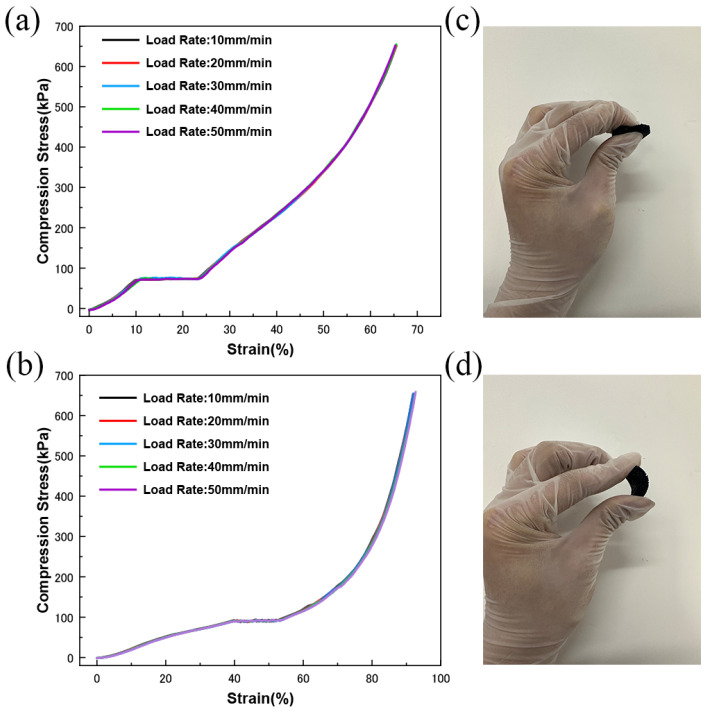
Mechanical properties of MCNT-PDMS/PDMS sponge sensors: (**a**) stress–strain curves under compressive cyclic loading; (**b**) stress–strain curve of pure PDMS sponge under compressive cyclic loading; (**c**) photograph of compressibility; (**d**) photograph of bendability.

To determine the effects of PDMS sponges with the addition of MCNT-PDMS coatings on the mechanical properties, we applied the same upper limit of force to both the pure PDMS sponges and MCNT-PDMS-coated sponges, releasing them at five different loading rates. Figure 3a,b display the classical stress–strain curves for the pure PDMS sponges and MCNT-PDMS/PDMS sponges. For both types of sponges, the mechanical response of the material was found to be independent of the applied load rate. The stress–strain curves at various loading rates completely overlapped, indicating that the material’s microstructure responds identically at all loading rates. At the same compressive strain, the MCNT-PDMS/PDMS sponges consistently experience higher pressure than the pure PDMS sponges, which can be attributed to the MCNT-PDMS coating, which enhances the mechanical properties of the sponges by providing additional support. The consistent mechanical behavior of the MCNT-PDMS/PDMS sponges and the improvement in their mechanical properties are crucial for the stability and operational range of the sensors. This suggests that any potential variations in piezoresistance at different loading rates are likely due to changes in the electrical behavior of the MCNT-PDMS composite network, rather than changes in the mechanical properties of the matrix material. It is also noteworthy that the MCNT-PDMS/PDMS sponges maintain excellent compressibility and bendability along with enhanced mechanical properties, as shown in Figure 3c,d.

**Figure 4:**
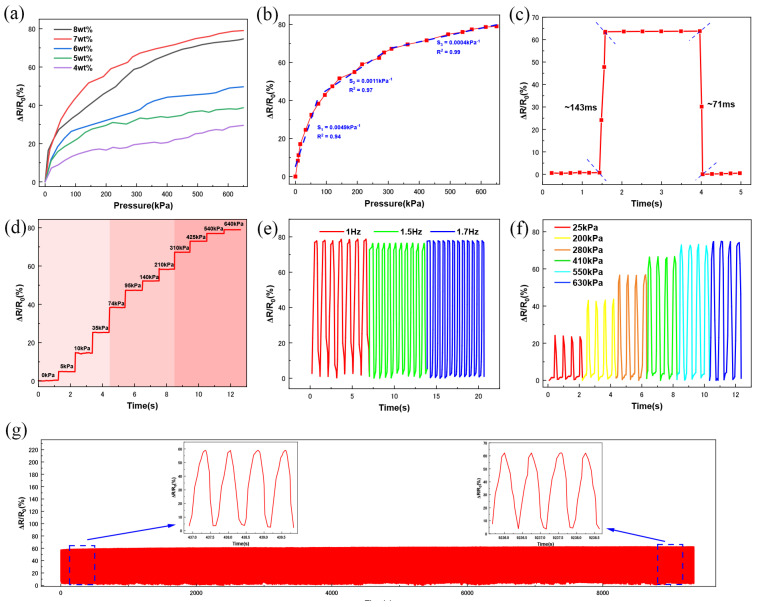
Sensing performance of MCNT-PDMS/PDMS sponge sensors: (**a**) relative change curves of resistance for MCNT-PDMS/PDMS sponge sensors coated with different MCNT-doped MCNT-PDMS at various compression states; (**b**) sensitivity curves of MCNT-PDMS/PDMS sponge sensors at different compression states; (**c**) response time and recovery time of MCNT-PDMS/PDMS sponge sensor; (**d**) response of MCNT-PDMS/PDMS sponge sensor under a step load from 0 kPa to 640 kPa; (**e**) response of MCNT-PDMS/PDMS sponge sensors at 600 kPa load at different frequencies (1 Hz, 1.5 Hz, and 1.7 Hz); (**f**) response of MCNT-PDMS/PDMS sponge sensors at different loads (25 kPa to 630 kPa) but the same frequency (1.8 Hz); (**g**) cyclic stability of MCNT-PDMS/PDMS sponge sensors over 10,000 compression cycles.

After preparing the entire sponge pressure sensor, we evaluated its sensing performance. Since the role of the conductive filler is to modulate the dielectric properties or resistance of the elastomeric sponge, the optimal ratio of the conductive filler is crucial in achieving high sensitivity in the device. By doping different mass fractions of MCNTs, we obtained PDMS sponges with MCNT-PDMS coatings (MCNT content by PDMS mass), with MCNT mass ratios ranging from 4 wt% to 8 wt%. A simple pressure sensor was assembled using MCNT-PDMS/PDMS sponges (with a 7 wt% MCNT mass fraction in the MCNT-PDMS coating) by connecting conductive nonwoven fabrics, which acted as electrodes, through a conductive silver paste for sensing performance evaluation. As shown in Figure 4a, the sensitivity of the MCNT-PDMS/PDMS sponges initially increased and then decreased. The highest sensitivity was observed at 7wt% MCNTs, while the decrease in sensitivity at 8 wt% can be explained by over-permeability theory, which describes the long-range connectivity formed by nodes or fillers in a stochastic system. When the polymer and conductive filler are blended, the conductivity of the composite gradually increases as the mass fraction of the conductive filler increases, reaching a maximum at the percolation threshold. According to over-permeability theory, the percolation threshold represents the maximum doping concentration before the conductive filler forms a continuous conductive path [43]. To determine the sensitivity of the pressure sensor, it was defined as

(1) $$S=\frac{\partial \left(\Delta R/R_{0}\right)}{\partial P}$$

where ΔR is the change in resistance when pressure is applied, R_0_ is the initial resistance of the sponge, and ΔP is the change in the applied pressure. As shown in Figure 4b, the MCNT-PDMS/PDMS sponge sensors can be categorized into three regions based on the sensing mechanism of the sensor: 0–74 kPa, 74–310 kPa, and 310–650 kPa, with sensitivities of 0.0049 kPa^−1^, 0.0010 kPa^−1^, and 0.0004 kPa^−1^, respectively, and linearities of 0.94, 0.97, and 0.98. These results indicate that the MCNT-PDMS/PDMS sponge-based pressure sensors have a wide operating range of up to 650 kPa, which is higher than that of some reported sponge pressure sensors [44,45,46]. The sensitivity of the sensor decreases gradually with increasing pressure, likely due to the gradual closure of the micropores, resulting in fewer conductive paths.

As shown in Figure 4c, when pressure of 280 kPa is applied, held, and then removed, the response time of the sensor is 143 ms, and the recovery time is 73 ms. Furthermore, a step load from 0 kPa to 640 kPa is applied to the sponge pressure sensor. Figure 4d shows the response of the sensor, which reflects the step characteristics. It can be observed that, as the pressure increases, the height of the step also increases, indicating the ability to accurately recognize different pressure levels across low, medium, and high pressure ranges. The sensor’s response to dynamic stimuli at different frequencies is also crucial for evaluation. As shown in Figure 4e, the relative change in resistance increases with increasing pressure, indicating that the sensor responds effectively to different pressures at the same frequency. The stability of the sensor suggests that it can function for extended periods. Figure 4f shows the sensor’s response at pressure of 600 kPa at frequencies of 1 Hz, 1.5 Hz, and 1.7 Hz. The results indicate that the sensor produces a similar peak output at different frequencies, with no distortion of the output signal as the frequency increases. As shown in Figure 4g, after more than 10,000 cycles of testing, the output signal remains stable with no drift, and the resistance change fluctuation is consistent after each loading–unloading cycle. From the two curves presented before and after cycling, it can be seen that the resistance of the sensor exhibits a cyclic pattern of change under dynamic pressure, with a rapid drop when pressure is applied and rapid recovery when the pressure is removed. Due to the elasticity and structure of the PDMS, the rebound resistance is higher than the stabilized resistance value but recovers in a short period of time. The sensor’s resistance decreases almost identically each time at a fixed intensity, direction, and frequency. When the sensor is repeatedly subjected to the same pressure, the peaks and valleys of the output values remain consistent, indicating high stability.

**Figure 5:**
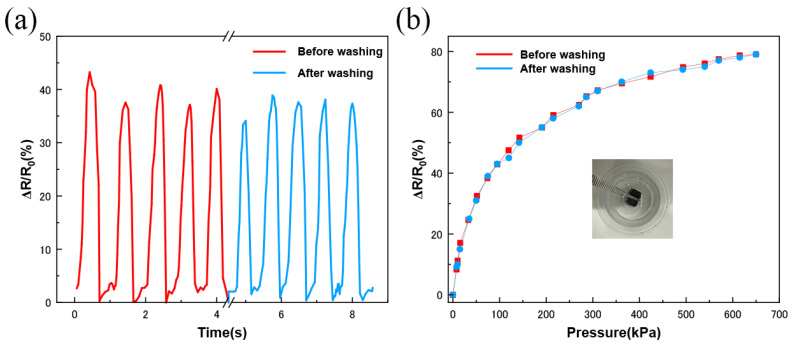
Washable performance test of MCNT-PDMS/PDMS sponge sensors: (**a**) relative resistance change curves before and after cleaning; (**b**) sensitivity curves before and after cleaning.

In addition, the MCNT-PDMS/PDMS sponge pressure sensor transducers were ultrasonically washed in deionized water for 2 h after 100 consecutive compressions, with no significant color change observed in the water. As shown in Figure 5a, there was no noticeable change in the peak output value of the sensor before and after washing at the same pressure. The sensitivity curves of the sensor at different strains before and after washing, shown in Figure 5b, almost completely overlapped. These results indicate that the sensor exhibits excellent stability during water washing. This is partly due to the strong adhesion formed by the MCNT-PDMS coating on the PDMS and partly due to the hydrophobicity of the cured PDMS surface [47,48].

**Figure 6:**
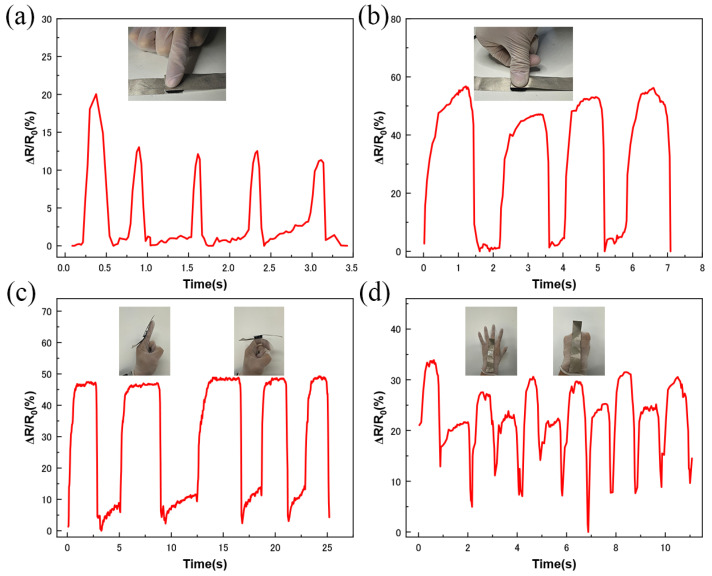
MCNT-PDMS/PDMS sponge sensors monitoring different hand movements in real time: (**a**) finger click; (**b**) finger press; (**c**) knuckle flexion; (**d**) five-finger extension.

The application of MCNT-PDMS/PDMS sponge pressure sensors in monitoring various human body activities was further investigated. First, we tested the sensors in the low-pressure range. As shown in Figure 6a, when the sensor is continuously clicked with a finger, the response curve clearly reflects the intensity and time interval of each click. Additionally, as shown in Figure 6b, when the sensor is pressed by a finger, the signal performance significantly differs from that of the click, with sharper peaks and a shorter duration for the finger click. This demonstrates that the sensor can distinguish between finger clicks and finger presses. The sensor was also fixed at the finger joints to detect finger bending movement. As shown in Figure 6c, when the finger is straight, the sensor’s resistance remains stable, while, when the finger is bent to a fixed angle, the resistance decreases accordingly, with the relative change in resistance becoming larger. Repeating the test multiple times yielded the same peak curve, indicating that the sensor can reliably detect finger bending. Similarly, we fixed the sensor on the back of the hand to monitor the five-finger stretching movement. As the fingers moved from open to a fist, they exerted a certain pressure on the sensor. Figure 6d shows the corresponding changes in the signal peak value, indicating that the sensor can differentiate between movements of varying amplitudes.

**Figure 7:**
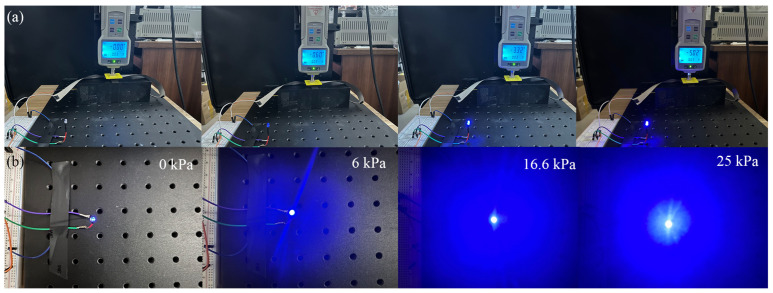
Resistance change in MCNT-PDMS/PDMS sponge sensors at different pressures, affecting the brightness of the LED: (**a**) test scenarios and the indication of the dynamometer at different pressures; (**b**) brightness response of the LED at different pressures ranging from 0 kPa to 25 kPa.

It is worth noting that the resistance of the sensor can be visualized through the brightness of the light-emitting diode (LED). As shown in Figure 7a, the LED and the sensor are connected in series with a constant voltage. Pressure is applied to the sensor via a high-precision displacement stage control dynamometer. Figure 7b shows that the LED becomes brighter as the pressure increases from 0 to 25 kPa.

**Figure 8:**
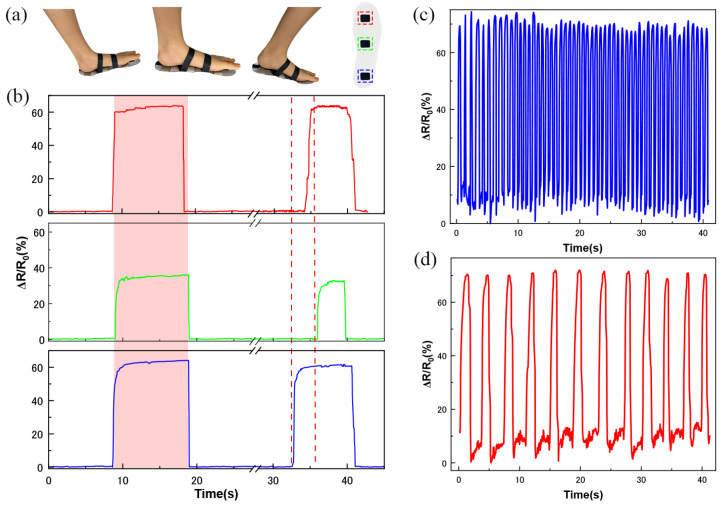
MCNT-PDMS/PDMS sponge sensors used to detect plantar pressure: (**a**) diagram of the walking process and the placement of the sensor on the insole; (**b**) relative resistance change curves at 3 points (forefoot(red line), arch(green line), and heel(bule line)) during walking; (**c**) relative resistance change curve for fast walking; (**d**) relative resistance change curve for slow walking.

Secondly, we tested the sensor’s application at medium pressures by monitoring real-time changes in plantar pressure. To monitor these changes, we placed pressure sensors in the forefoot, arch, and heel of the insole and wirelessly monitored the pressure on the sole via Bluetooth, as shown in Figure 8a. By analyzing the pressure at these three locations, we can monitor the motion of a normal foot while standing and walking (Figure 8b). When standing, the three sensors deform and produce resistance changes simultaneously, as shown in Figure 8b. The current change in the middle sensor is significantly smaller than that in the front and rear sensors, mainly due to the smaller deformation of the normal foot arch. Physiologically, the heel, arch, and ball of the foot land and leave in sequence during walking. The pressures at these three positions gradually increase and then decrease as the center of gravity moves forward. However, the sensor in the arch of the foot is the last to receive the pressure signal. This may be because the sensor in the arch position does not come into contact with the arch until the ball of the foot hits the ground. As the center of gravity moves forward, the pressure on the arch increases, causing the sensor to contact and deform. Meanwhile, we detected a person’s walking state by placing the sensors in the forefoot position. As shown in Figure 8c,d, the signal frequency for fast walking is significantly higher than that for slow walking. Through cyclic walking tests on the sole mold, the pressure response performance shows good agreement with the actual motion pattern. Once the foot’s pressure signal changes, the sensor can be monitored in real time. This plantar pressure monitoring can be used to analyze gait patterns and speeds, helping to assess athletic performance and rehabilitation.

**Figure 9:**
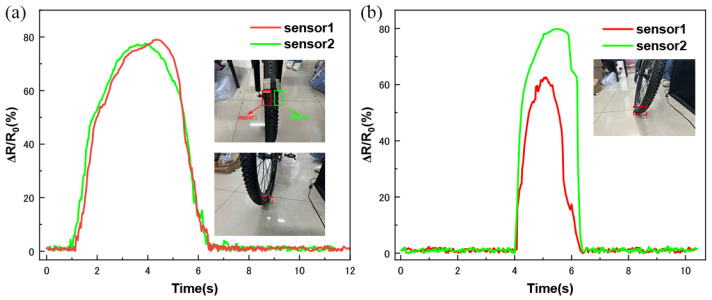
MCNT-PDMS/PDMS sponge sensors used to detect the riding state: (**a**) relative resistance change curve of dual channels for horizontal riding; (**b**) relative resistance change curve of dual channels for inclined riding.

Finally, to demonstrate the excellent toughness and wide dynamic pressure response of the MCNT-PDMS/PDMS sponge sensors, we conducted bicycle tire rollover experiments using dual-channel sensors placed at both ends of the bicycle tires, as shown in Figure 9a. The contours of the bicycle’s trajectory are clearly recorded in the pressure trajectories. As shown in Figure 9a, when riding horizontally, the sensors on both sides of the tire experience the same pressure, and the relative resistance change curves of the sensors on both sides overlap almost exactly. However, when riding at an incline, the sensors on the inclined side experience more pressure, which is clearly shown in Figure 9b, illustrating the difference in pressure received.

Professional cyclists use more riding tilt control and less steering control to achieve excellent balance performance at high speeds [49]. By monitoring the pressure differences between different sensors during riding, the tilt during steering can be reflected. These data can be used to optimize the cyclist’s steering posture at high speeds, improving their sports performance.

## 4. Conclusions

In conclusion, we used a simple sucrose sugar pill template to prepare PDMS sponges with a uniform pore size and distribution and applied MCNT-PDMS composites as conductive coatings to construct MCNT-PDMS/PDMS sponge sensors, which exhibited excellent performance. Since the conductive layer of the sensor is constructed from the same material as the substrate, it improves the adhesion of the conductive coating and enhances the mechanical properties of the sponge. The sensor exhibits a very wide operating range (0–650 kPa), excellent washability, long-term reproducibility (over 10,000 cycles), and a short response time (143 ms) and recovery time (71 ms), along with excellent stability. Based on these favorable characteristics, the MCNT-PDMS/PDMS sponge sensor can be used to monitor various stress states in daily life, such as gesture monitoring, plantar pressure monitoring, and cycling pressure monitoring. It has a wide range of applications in smart wearable health monitoring, robotic tactile sensing, and cycling pressure monitoring.

## Data Availability

The original contributions presented in this study are included in the article. Further inquiries can be directed to the corresponding author.

## References

[1] Wang X., Yu J., Cui Y., Li W. (2021). Research progress of flexible wearable pressure sensors. Sens. Actuators A Phys..

[2] Yuan Y., Liu B., Li H., Li M., Song Y., Wang R., Wang T., Zhang H. (2022). Flexible wearable sensors in medical monitoring. Biosensors.

[3] Qiu J., Liu S., Guo Y., Yang L., Jiang K. (2024). Anisotropic flexible pressure/strain sensors: Recent advances, fabrication techniques, and future prospects. Chem. Eng. J..

[4] Sinha A., Koo D., Kim J., So H. (2025). Physiologically compatible MWCNT-incorporated PNCPG self-healed ionic breathable hydrogel for wearable smart strain sensor application. Nano Today.

[5] Liu Y., Wang B., Chen J., Zhu M., Jiang Z. (2024). Flexible Nanofiber Pressure Sensors with Hydrophobic Properties for Wearable Electronics. Materials.

[6] Zhang X., Zheng J., Zhu Z., Hu C., Peng H., Liu B. (2024). Transparent, flexible nanoporous sensors with humidity and pressure sensing capabilities for sports health status monitoring. ACS Appl. Nano Mater..

[7] Li N., Liu J., Zhao S., Li Y., Wei Q. (2024). Skin-inspired bimodal pressure sensor for static and dynamic intelligent interactive perception system. Chem. Eng. J..

[8] Xu S., Xu Z., Li D., Cui T., Li X., Yang Y., Liu H., Ren T. (2023). Recent advances in flexible piezoresistive arrays: Materials, design, and applications. Polymers.

[9] Liu G., Lv Z., Batool S., Li M.Z., Zhao P., Guo L., Wang Y., Zhou Y., Han S.T. (2023). Biocompatible material-based flexible biosensors: From materials design to wearable/implantable devices and integrated sensing systems. Small.

[10] Lee H., Kwon D., Cho H., Park I., Kim J. (2017). Soft nanocomposite based multi-point, multi-directional strain mapping sensor using anisotropic electrical impedance tomography. Sci. Rep..

[11] Li W., Jin X., Han X., Li Y., Wang W., Lin T., Zhu Z. (2021). Synergy of porous structure and microstructure in piezoresistive material for high-performance and flexible pressure sensors. ACS Appl. Mater. Interfaces.

[12] Duan Z., Jiang Y., Huang Q., Wang S., Zhao Q., Zhang Y., Liu B., Yuan Z., Wang Y., Tai H. (2021). Facilely constructed two-sided microstructure interfaces between electrodes and cellulose paper active layer: Eco-friendly, low-cost and high-performance piezoresistive sensor. Cellulose.

[13] Zhao X., Zhao S., Zhang X., Su Z. (2023). Recent progress in flexible pressure sensors based on multiple microstructures: From design to application. Nanoscale.

[14] Li G., Chen D., Li C., Liu W., Liu H. (2020). Engineered microstructure derived hierarchical deformation of flexible pressure sensor induces a supersensitive piezoresistive property in broad pressure range. Adv. Sci..

[15] Zhang Y.X., He Y., Liang Y., Tang J., Yang Y., Song H.M., Zrínyi M., Chen Y.M. (2023). Sensitive piezoresistive pressure sensor based on micropyramid patterned tough hydrogel. Appl. Surf. Sci..

[16] Sun X., Sun J., Zheng S., Wang C., Tan W., Zhang J., Liu C., Liu C., Li T., Qi Z. (2019). A sensitive piezoresistive tactile sensor combining two microstructures. Nanomaterials.

[17] Qiu Y., Tian Y., Sun S., Hu J., Wang Y., Zhang Z., Liu A., Cheng H., Gao W., Zhang W. (2020). Bioinspired, multifunctional dual-mode pressure sensors as electronic skin for decoding complex loading processes and human motions. Nano Energy.

[18] Du D., Ma X., An W., Yu S. (2022). Flexible piezoresistive pressure sensor based on wrinkled layers with fast response for wearable applications. Measurement.

[19] Deng S., Zeng Q., Xiao Z., Zhang J., Yang G., Wu X., Li J., Zhang D., Zhou J., Liu B. (2025). High-performance flexible piezoresistive 3D pressure sensor based on wrinkled structures and porous microstructures. Mater. Lett..

[20] Lv Y., Zhang M., Zhao B., Qin Z., Chen K., Liu Y., Pan K. (2024). Flexible laser-reduced graphene with gradient-wrinkled microstructures for piezoresistive pressure sensors. ACS Appl. Nano Mater..

[21] Zhang P., Ang L., Li Y., Guo C., Zhang Y. (2024). Biomimetic Wearable Flexible Pressure Sensor with Multi-level Microstructured Piezoresistive Layer. IEEE Sens. J..

[22] Yan J., Ma Y., Jia G., Zhao S., Yue Y., Cheng F., Zhang C., Cao M., Xiong Y., Shen P. (2022). Bionic MXene based hybrid film design for an ultrasensitive piezoresistive pressure sensor. Chem. Eng. J..

[23] Guo X., Liu T., Tang Y., Li W., Liu L., Wang D., Zhang Y., Zhang T., Zhu X., Guan Y. (2024). Bioinspired low hysteresis flexible pressure sensor using nanocomposites of multiwalled carbon nanotubes, silicone rubber, and carbon nanofiber for human–computer interaction. ACS Appl. Nano Mater..

[24] Huang A., Gu S., Yang Z., Chen X., He M., Peng X. (2025). Flexible, Lightweight, and Hydrophobic TPU/CNT Nanocomposite Foam With Different Surface Microstructures for High-Performance Wearable Piezoresistive Sensors. J. Polym. Sci..

[25] Li Y., Jiang C., Han W. (2020). Extending the pressure sensing range of porous polypyrrole with multiscale microstructures. Nanoscale.

[26] Yin T., Cheng Y., Hou Y., Sun L., Ma Y., Su J., Zhang Z., Liu N., Li L., Gao Y. (2022). 3D Porous Structure in MXene/PANI Foam for a High-Performance Flexible Pressure Sensor. Small.

[27] Lee J., So H. (2023). 3D-printing-assisted flexible pressure sensor with a concentric circle pattern and high sensitivity for health monitoring. Microsyst. Nanoeng..

[28] Shin S., Ko B., So H. (2022). Structural effects of 3D printing resolution on the gauge factor of microcrack-based strain gauges for health care monitoring. Microsyst. Nanoeng..

[29] Wei H., Li X., Yao F., Feng X., Zhu X. (2024). Flexible piezoresistive pressure sensor based on a graphene-carbon nanotube-polydimethylsiloxane composite. Nanotechnol. Precis. Eng..

[30] Sun Q.J., Zhuang J., Venkatesh S., Zhou Y., Han S.T., Wu W., Kong K.W., Li W.J., Chen X., Li R.K. (2018). Highly sensitive and ultrastable skin sensors for biopressure and bioforce measurements based on hierarchical microstructures. ACS Appl. Mater. Interfaces.

[31] Bang J., Chun B., Lim J., Han Y., So H. (2023). Ultra-broad linear range and sensitive flexible piezoresistive sensor using reversed lattice structure for wearable electronics. ACS Appl. Mater. Interfaces.

[32] Bang J., Chun B., Kim M., Lim J., Han Y., So H. (2025). Rapid thermal runaway detection of lithium-ion battery via swelling-based state-of-charge monitoring using piezoresistive sponge sensor. eTransportation.

[33] Sun P., Wu D., Liu C. (2021). High-sensitivity tactile sensor based on Ti2C-PDMS sponge for wireless human–computer interaction. Nanotechnology.

[34] Sengupta D., Kamat A.M., Smit Q., Jayawardhana B., Kottapalli A.G.P. (2022). Piezoresistive 3D graphene–PDMS spongy pressure sensors for IoT enabled wearables and smart products. Flex. Print. Electron..

[35] Lee S.J., Ung S.C., Kim C.L. (2024). Highly compressible wearable sensor with CNT-coated PDMS sponge electrodes for tactile monitoring application. Phys. Scr..

[36] Ding Y., Yang J., Tolle C.R., Zhu Z. (2018). Flexible and compressible PEDOT: PSS@ melamine conductive sponge prepared via one-step dip coating as piezoresistive pressure sensor for human motion detection. ACS Appl. Mater. Interfaces.

[37] Ding Y., Xu T., Onyilagha O., Fong H., Zhu Z. (2019). Recent advances in flexible and wearable pressure sensors based on piezoresistive 3D monolithic conductive sponges. ACS Appl. Mater. Interfaces.

[38] Wang M., Zhang K., Dai X.X., Li Y., Guo J., Liu H., Li G.H., Tan Y.J., Zeng J.B., Guo Z. (2017). Enhanced electrical conductivity and piezoresistive sensing in multi-wall carbon nanotubes/polydimethylsiloxane nanocomposites via the construction of a self-segregated structure. Nanoscale.

[39] Cai Y., Liu L., Meng X., Wang J., Zhang C., Li J., Lu Z., Duan J.a. (2022). A broad range and piezoresistive flexible pressure sensor based on carbon nanotube network dip-coated porous elastomer sponge. RSC Adv..

[40] An Y., Liu J., Yan J., Feng H., Zhou R., Wu D., Yang J., Liu T., Sun J. (2025). A Simple and Efficient Preparation Method for Flexible Pressure Sensors Using Salt Template and Vacuum Infiltration. Adv. Mater. Technol..

[41] Zhang X., Dai H., Ji M., Han Y., Jiang B., Cheng C., Song X., Song Y., Wu G. (2025). A flexible piezoresistive strain sensor based on AgNWs/MXene/PDMS sponge. J. Mater. Sci. Mater. Electron..

[42] Kang S., Lee J., Lee S., Kim S., Kim J.K., Algadi H., Al-Sayari S., Kim D.E., Kim D., Lee T. (2016). Highly sensitive pressure sensor based on bioinspired porous structure for real-time tactile sensing. Adv. Electron. Mater..

[43] Mamunya Y.P., Muzychenko Y.V., Pissis P., Lebedev E., Shut M. (2002). Percolation phenomena in polymers containing dispersed iron. Polym. Eng. Sci..

[44] Wang Y., Luo W., Wen Y., Zhao J., Chen C., Chen Z., Zhang X.S. (2025). Wearable, washable piezoresistive pressure sensor based on polyurethane sponge coated with composite CNT/CB/TPU. Mater. Today Phys..

[45] Sheng M., Xiao Z., Deng S., Wu X., Zeng Q., Zhang J., Li J., Zhang D., Yang G., Qiang Q. (2025). A super water-stable polyaniline sponge sensor for multifunctional sensing of physical and chemical stimuli. Sens. Actuators A Phys..

[46] Trung V.D., Zhao W., Natsuki J., Tan J., Yang W., Natsuki T. (2025). Synergistic interfacial engineering for ultrasensitive bionic tunable strain sensors with robust sensing stability and integrated thermal management. Chem. Eng. J..

[47] Kallingal N., Kavil S.N., Fathima A., Ghosh P., Hussen E., Abdelrahman N., Ahmed R., Awad S., Kasak P., Popelka A. (2025). Fabrication of Bioinspired 3D-Printed Microtextures for Efficient Water Harvesting Using Plasma Treatment. J. Environ. Chem. Eng..

[48] Sheng S., Wang M., Mu L. (2025). An innovative and rapid method for permanent hydrophilic modification of polydimethylsiloxane (PDMS) chip surfaces. J. Appl. Phys..

[49] Cain S.M., Ashton-Miller J.A., Perkins N.C. (2016). On the skill of balancing while riding a bicycle. PLoS ONE.

